# Correction: Sleep-wake patterns are altered with age, Prdm13 signaling in the DMH and diet restriction in mice

**DOI:** 10.26508/lsa.202603771

**Published:** 2026-05-28

**Authors:** Shogo Tsuji, Cynthia S Brace, Ruiqing Yao, Yoshitaka Tanie, Hirobumi Tada, Nicholas Rensing, Seiya Mizuno, Julio Almunia, Yingyi Kong, Kazuhiro Nakamura, Takahisa Furukawa, Noboru Ogiso, Shinya Toyokuni, Satoru Takahashi, Michael Wong, Shin-ichiro Imai, Akiko Satoh

**Affiliations:** 1 Department of Integrative Physiology, National Center for Geriatrics and Gerontology (NCGG) , Obu, Japan; 2 Department of Developmental Biology, Washington University School of Medicine, St. Louis, MO, USA; 3 Department of Nutrition, Faculty of Wellness, Shigakkan University, Obu, Japan; 4 Department of Physiology, Yokohama City University Graduate School of Medicine, Yokohama, Japan; 5 Department of Neurology, Washington University School of Medicine, St. Louis, MO, USA; 6 Laboratory Animal Resource Center, University of Tsukuba, Tsukuba, Japan; 7 Laboratory of Experimental Animals, NCGG, Obu, Japan; 8 Department of Pathology and Biological Responses, Nagoya University Graduate School of Medicine, Nagoya, Japan; 9 Department of Integrative Physiology, Nagoya University Graduate School of Medicine, Nagoya, Japan; 10 Laboratories for Molecular and Developmental Biology, Institute for Protein Research, Osaka University , Osaka, Japan; 11 Department of Gerontology, Laboratory of Molecular Life Science, Institute of Biomedical Research and Innovation, Kobe, Japan; 12 Department of Integrative Physiology, Institute of Development, Aging, and Cancer, Tohoku University, Sendai, Japan

## Abstract

This study found that age-associated sleep changes are ameliorated by DR in the presence of Prdm13 signaling in the DMH, suggesting Prdm13+ DMH neurons will be of great interest to explore a potential intervention on age-associated sleep changes.

Article: Tsuji S, Brace CS, Yao R, Tanie Y, Tada H, Rensing N, Mizuno S, Almunia J, Kong Y, Nakamura K, Furukawa T, Ogiso N, Toyokuni S, Takahashi S, Wong M, Imai SI, Satoh A (2023 Apr 12) Sleep-wake patterns are altered with age, Prdm13 signaling in the DMH, and diet restriction in mice. Life Sci Alliance 6(6): e202301992. doi: https://doi.org/10.26508/lsa.202301992. PMID: 37045472.

Following publication, the authors were made aware that the methods for mouse experimentation involving sleep deprivation (SD) were not clearly described. Namely, the parameters used for the control setting, in which mice were permitted to sleep, were unclear in particular on food removal. We have updated the methods section “SD Study” to explicitly state that in the control setting, food was removed as in the treatment setting. We regret any confusion caused by the initial description in this section.

Corrected text:

SD Study

Mice were individually housed before the experiment. On the day of SD, food was removed at 6 am and mice were kept awake using a long Q-tip until 12 pm (6 h SD) by gentle touching of mice, as previously reported (Franken et al, 1991). Attempts to sleep were determined by the onset of behaviors typical of sleep such as cornering, curling, and eye closing. Once such behaviors were observed, we placed the long Q-tip in front of the mouse. One sleep attempt was counted when the mouse did not react to it. EEG/EMG recording was performed during SD to monitor the effectiveness of the SD protocol. All animals indicated <5% of sleep during the 6 h of SD. After SD, food was added, and the mice were allowed to sleep. Mice for control manipulation (AL-SD) were also individually housed before the experiment and during the experiment food was removed as in the SD group. We checked the level of blood glucose in the SD study and found that the level of blood glucose was indistinguishable between SD and AL-SD groups (126 ± 6 and 131 ± 4 mg/dl, respectively), revealing that nutritional status is equal between these two groups. Genotypes and conditions were blinded during the experimental procedures.

